# Late Transformation of a Dermatofibrosarcoma Protuberans Neck Sarcoma to a Neoplasm With Fibrosarcomatous Elements: Lessons Learned

**DOI:** 10.7759/cureus.30690

**Published:** 2022-10-26

**Authors:** Alexandros Poutoglidis, Evropi Forozidou, Antonios Skalias, Nikolaos Tsetsos, Paraskevi Karamitsou

**Affiliations:** 1 Department of Otorhinolaryngology-Head and Neck Surgery, 'George Papanikolaou' General Hospital, Thessaloniki, GRC; 2 Department of Otorhinolaryngology-Head and Neck Surgery, 424 General Military Hospital, Thessaloniki, GRC

**Keywords:** neoplasm, head and neck, fibrosarcoma, dermatofibrosarcoma protuberans, oncology

## Abstract

Dermatofibrosarcoma protuberans is a low-grade soft tissue sarcoma not often related to the head and neck area. Distant metastases are not often recorded. Wide surgical excision is considered the most appropriate treatment modality. In cases of insufficient or positive surgical margins, the role of adjuvant radiation therapy or targeted therapy might be beneficial. Dermatofibrosarcoma protuberans transformation to a neoplasm with fibrosarcomatous elements is extremely rare. The presence of fibrosarcomatous elements is associated with more aggressive behavior of the tumor and increases significantly its metastatic potential. We present a rare case of head and neck dermatofibrosarcoma protuberans with multiple local recurrences 13 years after the initial diagnosis.

## Introduction

Dermatofibrosarcoma protuberans (DFSP) is a rare low-grade soft tissue sarcoma with locally aggressive behavior [1]. Local recurrence is common, while distant metastases appear in up to 5% of cases. Although DFSPs are considered low-graded tumors, a small percentage of them have a solid fibrosarcoma component that increases significantly the metastatic potential of the neoplasm [2]. DFSPs are characterized by a uniform spindle cell arrangement and CD34 immunoreactivity [3]. However, the spindle cell morphology and CD34 immunoreactivity are reported in a broad range of other benign or malignant lesions. Derivation from fibroblasts in the dermis and subsequent infiltration into the subcutaneous tissues or direct development from subcutaneous tissues is seen histologically in DFSPs [3]. We present a rare case of a head and neck DFSP with multiple local recurrences 13 years after the initial diagnosis.

## Case presentation

**Figure 1:**
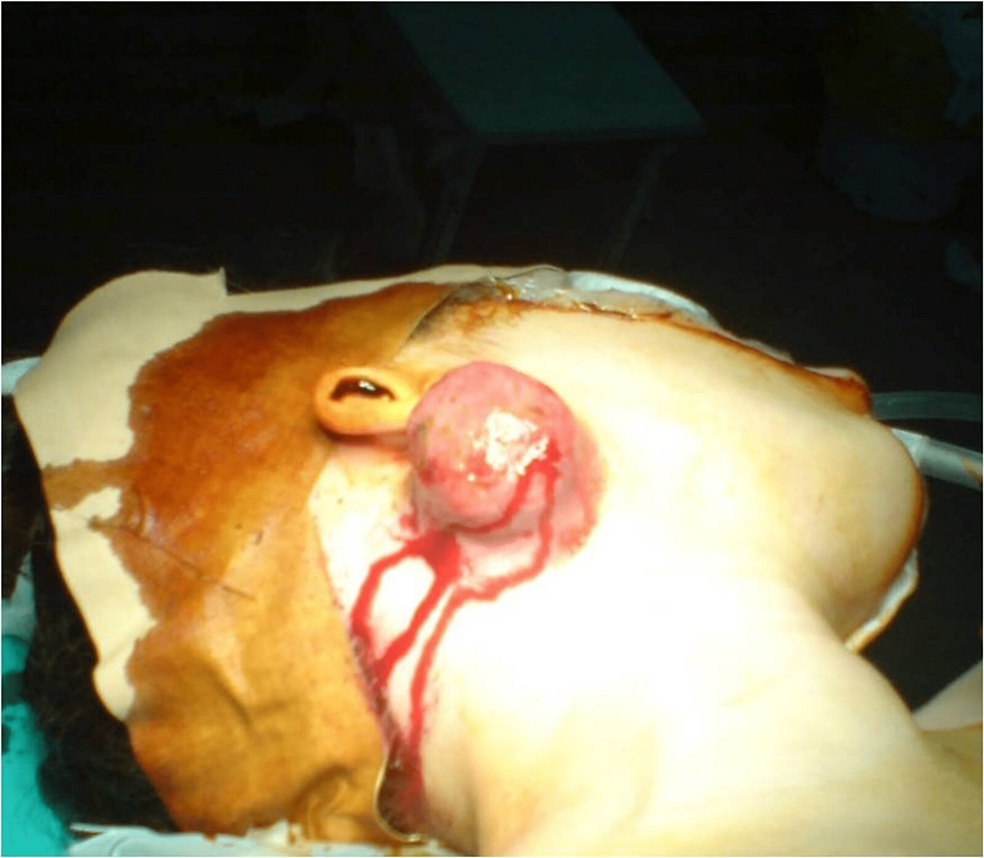
Tumor in the right parotid area An enlarged hemorrhagic tumor in the right parotid area. Patient's presentation 13 years ago.

A 40-year-old, non-smoker female presented to the Otolaryngology outpatient department with the chief complaint of a palpable mass on the right upper cervical region. No other accompanying symptoms were reported. The patient’s medical history included a previously excised DFSP in the right parotid area 13 years ago. A large hemorrhagic tumor appeared in the patient’s right parotid area at the age of 27 years old (Figure 1).

Magnetic resonance imaging (MRI) revealed a 3.7×2.9×2.6cm mass with homogeneous paramagnetic contrast agent uptake. A wide surgical excision was performed, including the right superficial parotid lobe with facial nerve preservation, the right submandibular gland, the right sternocleidomastoid muscle, the subcutaneous tissue, and the overlying skin. A local transpositional flap was recruited to cover the skin deficit. The histology report indicated a DFSP. Adjuvant radiation therapy followed the surgical excision. No recurrence was recorded in the patient’s follow-up appointments for 13 years.

At the time of its current presentation, a mass proximal to the area of the previous incision was reported 13 years later. Afterward, an MRI was requested to further evaluate the new mass, and a 1.4×2.0×2.6cm mass with heterogenous enhancement after intravenous paramagnetic contrast agent administration was revealed. Subsequently, wide surgical excision of the mass with the overlying skin was performed, concluding the diagnosis of a DFSP. The case was presented in the multidisciplinary team (MDT) meeting at our hospital. The decision of the MDT was to recommend adjuvant chemotherapy due to the tumor's recurrence and its histopathological characteristics.

**Figure 2:**
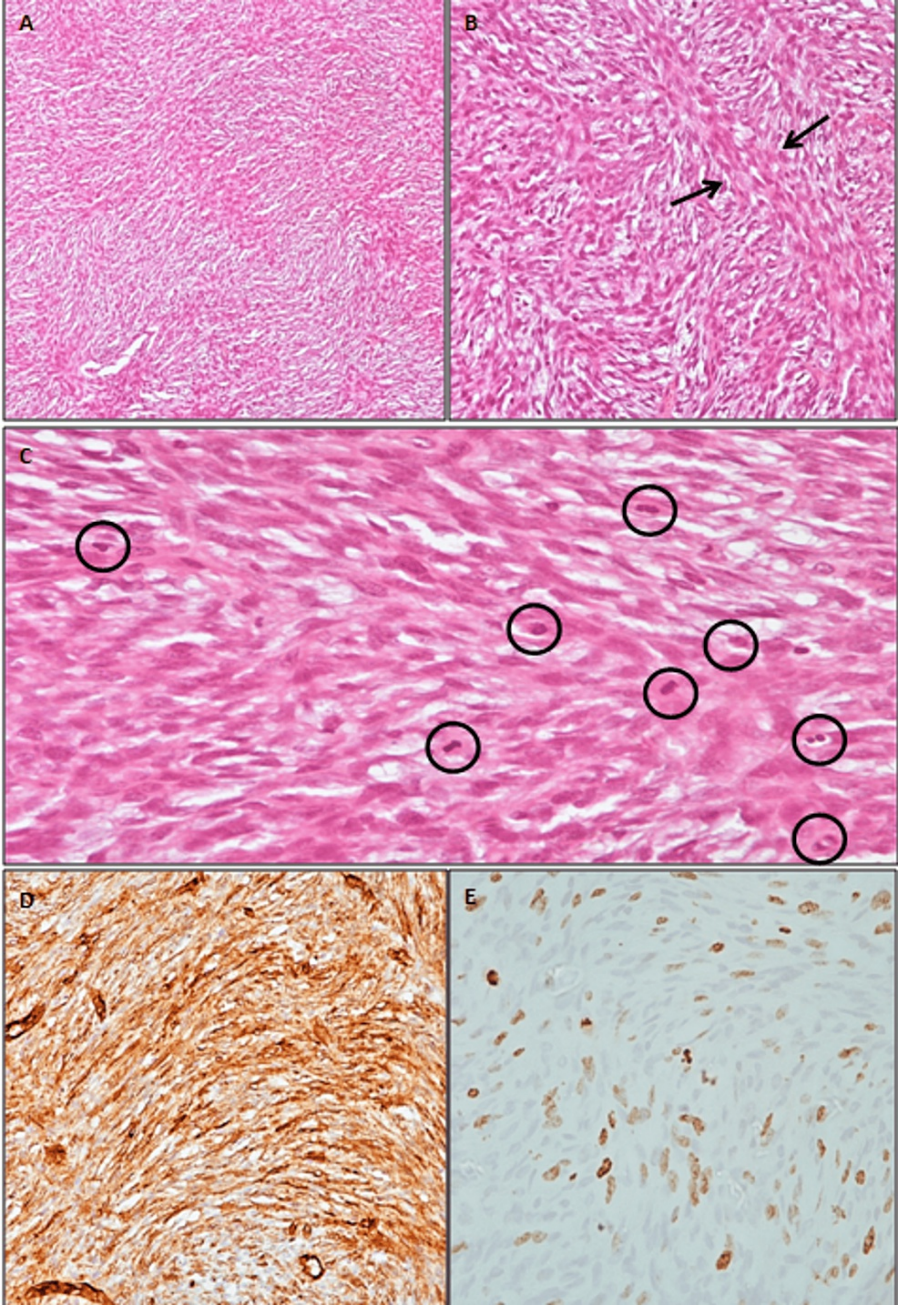
Histopathological characteristics A. The tumor was composed of spindled cells in a predominantly storiform whorled growth pattern. Spindled tumor cells containing plump or elongated wavy nuclei (hematoxylin and eosin staining (HE), 40×). B. Transition to a neoplasm with fibrosarcomatous elements (arrows) with atypia and mitosis (HE, 200×). C. Conventional dermatofibrosarcoma protuberans (DFSP) has <5 mitoses per 10 high-power field (HPF). In our case, high mitotic activity was recognized with >5 mitoses per 10 HPF (circles) (HE, 400×). D. Diffuse CD34 staining was positive (200×). E. Ki-67 proliferation index: 20%-25% (400×).

Ten months later, the presence of a palpable mass was detected in the same region and concomitant MRI revealed a well-enhanced mass of 3.9×2.1×2.7cm after intravenous paramagnetic contrast agent administration (Figure 2). Surgical excision of the tumor with the overlying cutaneous tissue was performed. The histology report indicated a malignant tumor with elements of a DFSP and areas with fibrosarcomatous elements (Figures 2A-2E).

Eight cycles of adjuvant platinum-based (cisplatin) chemotherapy were recommended for better local tumor control with no adverse side effects. Eleven months later, MRI was indicative of a 2.2×1.5cm round and well-enhanced mass. Subsequent surgical excision concluded in the diagnosis of a tumor with the previous histopathological characteristics. The MDT did not recommend adjuvant therapy taking also into account the low Ki-67 proliferation index (Ki-67: 3.5%). Twelve months after the last surgical procedure, the patient remains disease free. Meticulous follow-up every month is considered imperative for close recurrence surveillance due to the multiple recurrences.

## Discussion

DFSPs are rare low-grade and locally aggressive neoplasms that usually arise in the third decade of life [1]. Approximately 14% of cases affect the head and neck area. Patients with DSFP usually appear with a slowly enlarged thickening mass. Over the years, the tumor becomes firm and overlying telangiectasia is usually noticed [1]. The gold standard diagnostic tool is either incisional or core biopsy [4].

Magnetic resonance imaging is the modality of choice to evaluate the local tumor spreading after the initial diagnosis of DFSP with a biopsy. Soft tissue dissemination is accurately assessed, and a treatment plan can be determined. Ultrasound value is in the stage of diagnosis where it may guide targeted biopsies. Computed tomography better evaluates lymph node spread and possible bony infiltration. Finally, positron emission tomography is useful for the detection of distal metastasis [5].

Surgical excision with sufficient margins of 2cm to 4cm is considered the treatment of choice. Wide surgical margins are considered necessary due to the high likelihood of local recurrence in case of insufficient surgical margins [3]. Achievement of wide surgical margins in the head and neck area is often difficult to be performed. Thus, Mohs micrographic surgery is strongly recommended [3]. In cases of insufficient or positive surgical margins, DFSP is considered refractory to conventional chemotherapy the patient might benefit from adjuvant radiation therapy or targeted therapy with imatinib, a tyrosine kinase inhibitor after the discovery of the translocation t(17;22)(q22;q13) (COL1A1;PDGFB) in the vast majority of patients [6]. Surgical excision in the head and neck area poses risks to the integrity of the facial nerve due to the complexity of its pattern, and a nerve monitoring should be considered to avoid major complications [7].

If DFSPs are left untreated, the neoplasm demonstrates locally infiltrative invasiveness of the adjacent tissues, including fascia, muscular tissue, neurovascular bundles, and even periosteum [1]. Local recurrences in the lymph nodes are the most common pattern of metastasis; however, a lung metastasis may be present without lymph node involvement. Untreated neoplasms are known to infiltrate the fat, fascia, and local muscles. Bone infiltration of the head and neck area is extremely rare [1]. The exophytic nature of the DFSP and the slow rate of growth seem to be the main factors that restrict the bone infiltration. Additionally, the periosteum forms a solid barrier that resists tumor extension to the surrounding bones.

DSFP transformation to a neoplasm with fibrosarcomatous elements is extremely rare. The presence of foci with fibrosarcomatous elements in a DSFP tumor increases significantly the metastatic potential [8]. According to Mentzel et al, 15% of patients with fibrosarcomatous transformation develop pulmonary metastases [9].

## Conclusions

DFSP is a rare soft tissue sarcoma with locally aggressive behavior. A timely diagnosis and treatment require high clinical suspicion. Wide surgical excision is reported to be the most appropriate treatment method. The achievement of wide surgical margins diminishes the rate of recurrence rate and offers a better quality of life. More studies are required to clarify the role of radiation therapy or targeted therapy in treatment. Frequent local recurrences of the neoplasm make meticulous follow-up imperative for recurrence surveillance.
